# Enhanced Electrical Conductivity of Carbon Nanotube-Based Elastomer Nanocomposites Prepared by Microwave Curing

**DOI:** 10.3390/polym11071212

**Published:** 2019-07-25

**Authors:** Blake Herren, Preston Larson, Mrinal C. Saha, Yingtao Liu

**Affiliations:** 1 School of Aerospace and Mechanical Engineering, University of Oklahoma, Norman, OK 73019, USA; 2 Samuel Roberts Noble Microscopy Laboratory, University of Oklahoma, Norman, OK 73019, USA

**Keywords:** microwave irradiation, polydimethylsiloxane, carbon nanotubes, electrical conductivity, microstructure, nanoparticle distribution, alignment

## Abstract

Nanocomposites consisting of polydimethylsiloxane (PDMS) and well-dispersed carbon nanotubes (CNT) can be cured by microwave radiation within a minute, forming a conductive network within the cured materials. Microwave irradiation delivers energy directly to the inner core of the nanocomposites by heating CNTs and initiating rapid polymerization of the elastomer. In this paper, nanocomposites were fabricated with CNT loadings between 0.5 wt.%–2.5 wt.% via microwave irradiation. Key properties of the nanocomposites including electrical conductivity, microstructures, CNT distribution, density, and surface effects were all characterized. The properties of microwave-cured nanocomposites were compared with those manufactured by the thermal method using a conventional oven. The microwave-curing method substantially increased the electrical conductivity of the nanocomposites due to the improved nanoparticle dispersion and likely CNT alignment. Optimal microwave-curing parameters were identified to further improve the conductivity of the nanocomposites with lowest CNT loading. A conductivity enhancement of 142.8% over thermally cured nanocomposites was achieved for nanocomposites with 1 wt.% CNTs cured via one-step microwave irradiation.

## 1. Introduction

Electrically conductive nanoparticles have been widely used in recent years as fillers in polymers to create conductive nanocomposites with versatile behavior for a wide variety of applications. Commonly used conductive nanoparticles including silver nanowires [1], gold nanowires [2], graphene [3], carbon black [4], carbon nanofibers (CNFs) [5,6,7], and carbon nanotubes (CNTs) [8,9,10] are either randomly dispersed within a polymer matrix or well-aligned to fabricate nanocomposites with a wide variety of functions. Due to the outstanding electrical, thermal, and mechanical properties of these nanoparticles, they have been implemented to develop nanocomposites for applications including highly-flexible strain sensors [11], electronic skins [12], biomedical devices [13], and electromagnetic shielding [14]. Of these electrically conductive nanoparticles, CNTs have received widespread attention for their impressive properties. When CNTs are dispersed within a polymer matrix, their quality of dispersion and alignment have a substantial impact on the properties that they endow on the nanocomposite. Many different polymers have been used as matrix materials with dispersed CNTs to fabricate nanocomposites with improved mechanical properties [15], thermal conductivity [16], and electrical conductivity [17,18]. Substantial attention has been placed on improving the dispersion or controlling the alignment of these high aspect ratio nanoparticles, particularly for their use in electrically conductive nanocomposites.

In-situ alignment techniques include methods to achieve CNT alignment during the growing process, while ex-situ alignment techniques are implemented during the integration process of the CNTs [19]. Ex-situ alignment techniques include the use of electric fields [20,21], magnetic fields [22], surface forces [23], acoustic waves [24], mechanical stretching [25], and extrusion-based methods such as three-dimensional (3D) printing [26]. These techniques have proved useful to improve the CNT alignment in a chosen direction, often significantly enhancing the electrical conductivity of the nanocomposite in the aligned direction of the heterogeneous microstructures [27]. Numerous efforts have focused on improving the conductivity of one-dimensional carbon nanoparticle-enhanced nanocomposites including adding secondary conductive nanofillers such as carbon black [28], melt annealing of CNF-based thermoplastic nanocomposites [29], and investigating the impact of CNT waviness [30], aspect ratio [31,32], and agglomerate content [31,33] on electrical conductivity. While these studies have proven useful, not enough work has been done to explore the influence of rapid microwave curing of nanocomposites containing dispersed CNTs on the materials’ electrical conductivities.

Microwave curing has emerged in recent years as an efficient method to cure composite laminates [34,35,36], thermoset resins [37], and has been proven beneficial in curing CNT doped epoxies with significantly reduced curing times and energy consumptions [38]. Due to their high microwave absorption performance, carbonaceous nanoparticles can be easily heated via microwave irradiation [39]. When exposed to microwave energy, CNTs experience significant heating effects that, when dispersed well within an uncured thermoset matrix, transfer localized heat to the matrix material inducing rapid polymerization of the nanocomposite. The mechanisms involved with microwave-induced heating of CNTs are not fully understood; however, the current explanation includes the CNTs transferring electromagnetic energy to mechanical vibrations and impurities in the nanoparticles producing Joule heating when subject to microwave irradiation [40,41]. Chang et al. most notably found that microwave curing of CNT-based epoxies resulted in alignment in some direction, proven by observing the significant difference in CNT pullout on two vertical fractures of their microwave-cured nanocomposites, compared to no differences seen in their oven-cured samples [42]. In addition to CNT alignment induced by microwave irradiation, rapid polymerization via microwave curing decreases the settlement effect of the CNTs improving the resulting dispersion within the cured nanocomposite. Both dispersion and CNT alignment play a vital role in the electrical conductivity of nanocomposites; therefore, it is essential to examine the effects of microwave curing on the electrical conductivity of these materials. While a notable number of studies have been done to investigate rapidly curing CNT-based epoxies via microwave irradiation [38,42,43,44,45,46], little work has been done to explore microwave curing of CNT-based elastomers.

In this paper, we investigated the effects of microwave irradiation on the electrical conductivity of polydimethylsiloxane (PDMS) nanocomposites containing dispersed multi-walled CNTs. Cylindrical nanocomposites were fabricated using both microwave irradiation and thermal curing using a conventional oven. During the microwave curing procedure, temperature measurements were taken between each pulse of the microwave to investigate the rate of temperature increase. Next, the lowest conductive loading of CNTs was selected for further investigation of microwave curing parameters that improve the dispersion and alignment of the CNTs and consequently enhance the electrical conductivity of the nanocomposites. After the optimal microwave parameters were established for curing the lowest loading conductive material, a final conductivity comparison was made with the thermal curing method again to quantify the improvement of bulk conductivity made from the microwave curing optimization process.

## 2. Materials and Methods

### 2.1. Materials

All materials were used as received from the suppliers unless otherwise stated. The tetrahydrofuran (THF) used for the solvent-based nanoparticle dispersion method was purchased from Sigma Aldrich (St. Louis, MO, USA). The SYLGARD 184 PDMS kit is a two-part PDMS including the base elastomer (part A) and curing agent (part B) which was purchased from Dow Corning (Freeland, MI, USA). The multi-walled CNTs were purchased from Sigma Aldrich and had a diameter between 50–80 nm and an aspect ratio >100.

### 2.2. Nanoparticle Dispersion

In order to sufficiently disperse the conductive nanoparticles throughout the elastomeric matrix, a solvent-based ultrasonication method was used. First, a predetermined amount of CNTs were mixed in 30 mL of THF with a magnetic stir bar at 350 rpm for 5 min to wet the nanoparticles. Then, the CNT/THF mixture was sonicated with a 750-watt probe tip sonicator for 10 min to break apart the CNT agglomerates. Concurrently, a predetermined amount of the PDMS part A and 20 mL of THF were mixed with a magnetic stir bar at 350 rpm for 3 min to fully dissolve the base elastomer and lower the viscosity to allow for successful nanoparticle dispersion. Next, the CNT/THF mixture was added to the PDMS/THF solution and mixed with a stir bar at 350 rpm for 5 min. This mixture was sonicated for 30 min to successfully disperse the CNTs within the PDMS part A. Then, the PDMS/CNT/THF mixture was mixed with a magnetic stir bar at 350 rpm on a 70 °C hot plate to evaporate most of the THF. Once the stir bar was no longer able to rotate due to the decreased amount of THF and high viscosity of the nanocomposite resin, the stir bar was removed, and the mixture was placed in a vacuum oven at 70 °C overnight to completely remove residual THF. Each batch of material prepared resulted in 15 g of PDMS/CNT resin including only the PDMS part A and dispersed CNTs.

### 2.3. Nanocomposite Sample Fabrication

**Figure 1:**
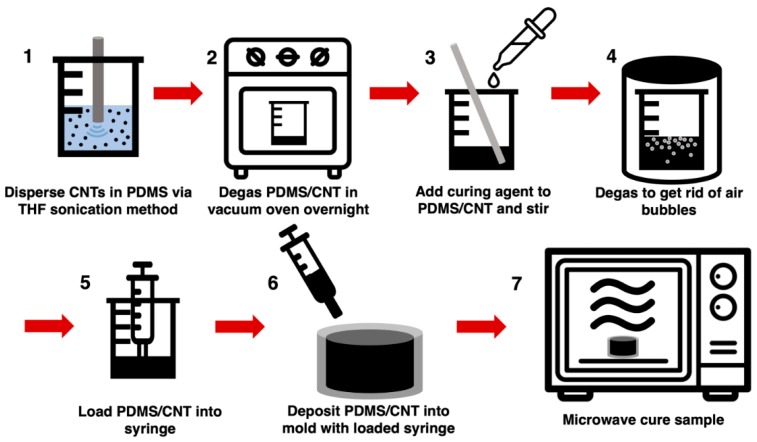
Schematic of sample fabrication method for m-PDMS/CNT.

The cylindrical microwave-cured PDMS/CNT samples (m-PDMS/CNT) were fabricated using the method described in the schematic shown in Figure 1. After the CNTs were dispersed in the PDMS base elastomer using the solvent-based dispersion method described above, the PDMS/CNT was removed from the oven and allowed to cool to room temperature before adding the PDMS part B to the nanocomposite at a 1:10 curing agent to base polymer ratio. The curing agent was thoroughly hand-mixed into the nanocomposite resin for 5 min and then degassed in a vacuum chamber for 1 h to remove air bubbles. Then, the nanocomposite prepolymer was loaded into a 3 mL syringe either through the open tip of the syringe with the plunger for lower viscosity resins, or the plunger was removed, and the higher viscosity resins were loaded in the larger back opening of the syringe. Next, the cylindrical glass mold (diameter = 11 mm, height = 9 mm) was filled with the PDMS/CNT prepolymer using the syringe, then the top surface was smoothed with a straight edge. The uncured nanocomposite resin in the glass mold was placed in the center of an unmodified 2.2 cubic feet General Electric 1200-watt microwave and cured via microwave irradiation at a preset power level, time, and number of cycles. Many of the samples experienced a small amount of deformation above the top surface of the mold due to thermal shock; therefore, the uneven top surface of each sample was removed with a razor blade to create a flat surface.

### 2.4. Density Measurements and Pore Imaging

The volume of each sample was determined by measuring the diameter and height of each cylindrical sample with a caliper and micrometer. The mass of each sample was measured with a digital scale then the density of each sample was calculated with these measurements. The porosity within the lowest and highest loading of conductive m-PDMS/CNT samples (1 wt.% and 2.5 wt.%) were imaged using a Zeiss Neon EsB scanning electron microscope (SEM) on the surface where the expanded material was removed with a razor blade to characterize the size of the pores remaining in the samples. The samples were sputter coated with approximately 5 nm of AuPd to reduce charging artifacts and aid in imaging.

### 2.5. Comparison of Microwave and Thermal Curing Methods

An aluminum mold was manufactured with a CNC milling machine to approximately the same size and shape as the glass mold used for microwave curing. To compare the conductivity effects of the microwave curing method and traditional oven curing of the PDMS/CNT nanocomposite, five batches of material with loadings between 0.5 wt.%–2.5 wt.% were prepared. Each batch of material was used to fabricate four thermally cured PDMS/CNT samples (t-PDMS/CNT) and four m-PDMS/CNT samples. During the mold loading procedure, one loaded syringe was used to fill one glass mold and one aluminum mold with PDMS/CNT prepolymer, alternating the order to ensure a fair comparison. The aluminum mold containing all four samples was placed in a 150 °C oven right before beginning to fabricate the m-PDMS/CNT samples one at a time. The oven curing times for the t-PDMS/CNT samples for 0.5 wt.%, 1 wt.%, 1.5 wt.%, 2 wt.%, and 2.5 wt.% loadings of the nanocomposite were 30 min, 1 h, 3 h, 5 h, and 7 h respectively. The microwave was run at 20% power and pulsed on for a variable amount of time depending on the loading of CNTs and pulsed off for 10 s to take temperature readings with a Fluke Ti25 Thermal Imager. The number of pulse cycles and time on per cycle to fabricate the m-PDMS/CNT samples for 0.5 wt.%, 1 wt.%, 1.5 wt.%, 2 wt.%, and 2.5 wt.% loadings of the nanocomposite were four pulses of 120 s, three pulses of 30 s, three pulses of 25 s, three pulses of 20 s, and four pulses of 15 s, respectively.

The resistance of each sample was first measured using an Instron 3345 single column mechanical testing machine and an Agilent 34401a multimeter. Each sample was placed in between two copper plates soldered to wires connected to the multimeter, and a 2N load was applied in compression to ensure complete contact with the nanocomposite sample. The resistance was averaged over 1 min after the measurement had adequately stabilized. Next, a layer of silver epoxy and copper tape was applied to each flat surface of the nanocomposite samples and allowed to harden. The resistance of the sample excluding the contact resistance between two copper plates was measured by clamping both extended ends of the copper tape to the multimeter and waiting for the resistance measurement to stabilize before averaging over 1 min. The reported contact resistance was the first measured resistance between the copper plates subtracted by the second measured resistance using the attached silver epoxy and copper tape. The conductivity of each sample was calculated from the resistance measured with the silver epoxy and copper tape.

To inspect the quality of nanoparticle dispersion in the nanocomposites, pore sizes and shapes, and surface topography as a result of the two curing methods, SEM imaging was performed on multiple samples. The nanoparticle dispersion within the lowest loading and highest loading of conductive m-PDMS/CNT samples (1 wt.% and 2.5 wt.%) were imaged on the surface where the expanded material was removed with a razor blade. The topography of the bottom surface of 1 wt.% m-PDMS/CNT and 1 wt.% t-PDMS/CNT were imaged to characterize the surface impact of the glass and aluminum molds.

### 2.6. Optimization of Microwave Curing Parameters

In order to investigate the optimal microwave settings for producing the highest conductivity m-PDMS/CNT samples with the lowest loading, two studies were performed varying the number of pulse cycles and the power of the microwave used to cure the samples. For each study, one batch of 1 wt.% PDMS/CNT resin was prepared, as this was the lowest conductive loading of material found during the initial curing method comparison.

The pulse study was performed using a constant microwave power of 50% and 10 s off was allowed between each pulse on to take temperature measurements. After filling three glass molds with uncured PDMS/CNT prepolymer, the m-PDMS/CNT samples were cured for eight pulses of 5 s, three pulses of 10 s, and one pulse (one-step) of 20 s. This process was repeated four times producing a total of 12 samples with about 15 min between the fabrication of each set of samples.

The power study was completed using one-step microwave irradiation at microwave powers of 20%, 50%, and 80%. Again, 12 total samples were prepared including one sample per power level in each set of samples and about 15 min between the fabrication of each set. Each sample from both the pulse study and power study were tested for their bulk conductivity using silver epoxy and copper tape applied to both flat ends of the cylindrical nanocomposites.

To determine the maximum increase of conductivity using the optimal microwave curing settings for the lowest conductive loading of PDMS/CNT, one batch of 1 wt.% PDMS/CNT was prepared. The optimal microwave power and pulse settings (one-step at 50% power) were used to cure four samples of m-PDMS/CNT while simultaneously curing four t-PDMS/CNT samples in the oven at 150 °C for 1 h. During the loading procedure into the molds, one loaded syringe was used to fill one glass mold and one aluminum mold alternating the order for each set of samples. The conductivity of each prepared sample was measured using silver epoxy and copper tape applied on both flat ends of the nanocomposites.

## 3. Results and Discussions

### 3.1. Evaluation of CNT Dispersion Quality

**Figure 2:**
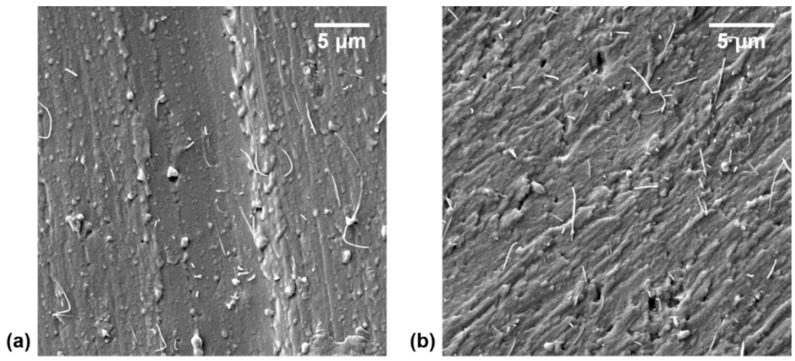
Scanning electron microscope (SEM) images of CNT dispersion within (**a**) 1 wt.% and (**b**) 2.5 wt.% m-PDMS/CNT.

The quality of CNT dispersion within a polymeric matrix greatly affects the conductivity and the microwave curing process of the nanocomposite. Agglomerates are known to superheat when exposed to microwave irradiation due to their high concentration of CNTs and can lead to localized thermal degradation of the surrounding polymer [38]. Notably, better dispersion of CNTs within a matrix can lead to higher microwave absorption of the nanocomposite but is not inevitably the case [47]. In addition, better CNT dispersion is generally assumed to offer higher conductivities of the nanocomposite, though this is not always true [17,32,48]. The best dispersion of CNTs in a matrix would result in a polymer layer around each CNT, thus shear-induced reaggregation [49] and solvent-based spinning techniques [50,51,52] have proven to have the ability to increase the conductivity of a nanocomposite even though the overall quality of CNT dispersion was reduced [17]. Regardless, it is imperative that the quality of dispersion within the m-PDMS/CNT nanocomposites be investigated to confirm consistent and high-quality CNT dispersion was achieved. The SEM images of the cut surface of 1 wt.% and 2.5 wt.% m-PDMS/CNT samples are shown in Figure 2a,b, respectively, verifying that the nanocomposite dispersion method was successful. No agglomerates were seen during the SEM investigation of the samples.

### 3.2. Investigation of Density and Porosity

**Figure 3:**
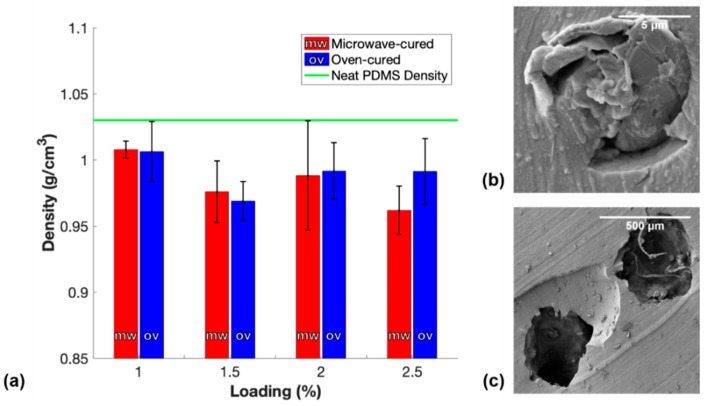
(**a**) Density measurements of conductive m-PDMS/CNT and t-PDMS/CNT samples, (**b**) SEM image of a pore within 1 wt.% m-PDMS/CNT, and (**c**) SEM image of pores within the lowest density 2.5 wt.% m-PDMS/CNT sample.

Undesired porosity is a common issue when manufacturing elastomeric thermosets. In general, the porosity of cured nanocomposites is calculated by:

(1) $$P=1-\frac{d_{f}}{d_{s}}$$

where *d_f_* is the density of manufactured nanocomposites and *d_s_* is the density of solid nanocomposites. In this study, mixing the curing agent into the PDMS/CNT resin during the manufacturing process introduced a large number of air bubbles within the polymer, which led to the need to degas the mixture in a vacuum chamber to remove the trapped air. It was observed that nanocomposites with CNT loadings of 1.5 wt.%, 2 wt.%, and 2.5 wt.% did not show any signs of releasing air bubbles during the degassing process due to thixotropic effects endowed on the resin by the reinforcing nanofiller. Furthermore, additional air bubbles were likely to form during the loading procedures into the 3 mL syringe and into the molds. Because porosity impacted the average cross-sectional area of the samples which could consequently impact the measured conductivity of the sample, it was necessary to measure the density of each sample and report any porosity. Figure 3 shows the average densities of each conductive loading of both the m-PDMS/CNT and t-PDMS/CNT samples.

The average density of each set of samples falls below the supplier’s reported density of PDMS; therefore, there was some amount of porosity in each sample. Notably, the PDMS/CNT resins with a loading of 1.5 wt.% and higher had a lower average density than the 1 wt.% resin. Although a higher loading of CNTs in the elastomer should result in a slightly higher density sample, the superior yield strength of the viscoelastic resin prevented air bubbles from releasing during degassing. Moreover, the materials with a higher CNT loading were more difficult to load in the syringe and into the mold without introducing additional embedded pores. The SEM image of a small pore within 1 wt.% m-PDMS/CNT seen in Figure 3b was likely formed as a result of either loading the material in the syringe or into the mold. The larger pores within the lowest density 2.5 wt.% m-PDMS/CNT sample seen in Figure 3c were likely a result of the inability to degas the material. Generally, the densities between oven-cured samples and microwave-cured samples for the same loading were comparable; therefore, the relative overall porosities of the samples were determined to be negligible in their impact on the conductivity measurements of the samples.

### 3.3. Comparison of Curing Methods

**Figure 4:**
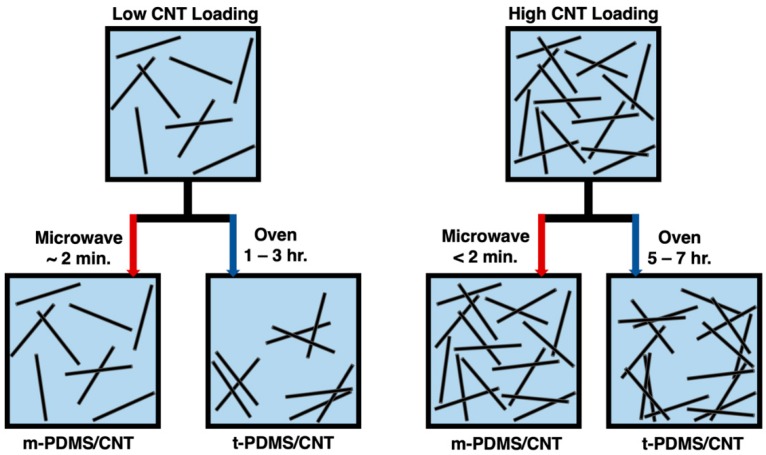
Schematic of increased dispersion for m-PDMS/CNT due to reducing CNT settlement effect expected in t-PDMS/CNT for low and high CNT loadings.

The percolation curve of both m-PDMS/CNT and t-PDMS/CNT was investigated due to the impact on conductivity we expected in terms of the significantly faster curing times reducing the settlement effect of the conductive nanoparticles. The settlement effect within CNT doped polymers was a result of gravity and van der Waals forces attracting the nanoparticles together reforming agglomerates and reducing the overall quality of dispersion. It was observed that this effect had a negative impact on the overall conductivity of the nanocomposites simply due to fewer opportunities for conductive networks to form. A schematic of this nanoparticle settlement effect for low and high loadings of CNTs is shown in Figure 4.

**Figure 5:**
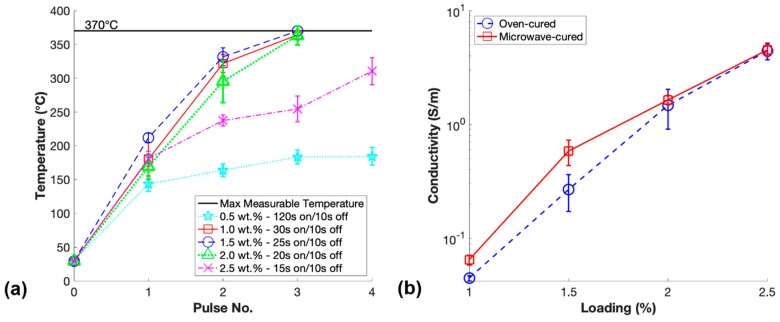
(**a**) Average temperature measurements between pulses of m-PDMS/CNT for each loading prepared and (**b**) conductivity averages for each loading of m-PDMS/CNT and t-PDMS/CNT.

Additionally, there have been reports that the microwave curing of CNT doped epoxies likely leads to the alignment of these high aspect ratio nanoparticles in some direction [42]. The alignment of these conductive nanoparticles within the matrix material may provide a second mechanism to increase the overall conductivity of the microwave-cured nanocomposites. Therefore, it was essential to investigate the conductivity improvements that could be obtained through microwave curing the same batch of PDMS/CNT resin at different loadings, reducing the loading at an increment of 0.5 wt.% starting at 2.5 wt.%. Furthermore, it was important to measure the temperature of each sample during the microwave curing process to aid future researchers in the microwave curing of PDMS/CNT or materials with similar CNT content. The temperatures between pulses of each loading of prepared m-PDMS/CNT and the percolation curves for both m-PDMS/CNT and t-PDMS/CNT are shown in Figure 5.

The maximum measurable temperature was included in Figure 5a, as this was the maximum measurable temperature of the thermal imager, and during the last step of curing 1.5 wt.% m-PDMS/CNT the temperature reached slightly above this threshold. Progressively shorter pulsing times of 20% microwave power were chosen for higher loading material to prevent the thermal shock expansion of the material from ripping the samples apart, which was observed if the temperature rose too quickly. Increments of 5 s were chosen to make the pulsing cycles simple to consistently achieve manually pulsing the microwave at the correct times. Interestingly, the nonconductive 0.5 wt.% PDMS/CNT required a significantly longer curing time of 4 pulses of 120 s due to a lack of conductive networks in the resin to aid in the microwave heating process. Although 2.5 wt.% m-PDMS/CNT had the highest conductivity due to having the highest loading of materials tested, the rate of temperature change was the slowest of the conductive resins due to the relatively short time of 15 s used for each pulse of the microwave.

The percolation curves for both m-PDMS/CNT and t-PDMS/CNT shown in Figure 5b indicate that for each conductive loading, the microwave-cured samples had a higher conductivity than the oven-cured samples. The average percentage increase of conductivity for m-PDMS/CNT over t-PDMS/CNT for loadings 1 wt.%, 1.5 wt.%, 2 wt.%, and 2.5 wt.% were 44.8%, 118%, 11.7%, and 1.8%, respectively. At higher loadings, 2 wt.% and 2.5 wt.%, the conductive networks were more saturated, thus, the impact of the CNT settlement effect on the decrease of conductivity was lesser, illustrated by the schematic shown in Figure 4. Additionally, any microwave-induced CNT alignment effects that generated an increase in conductivity were also likely less impactful in nanocomposites with higher loadings of CNTs. Surprisingly, the increase of conductivity due to microwave curing in this study was significantly more dramatic for the 1.5 wt.% PDMS/CNT. This indicated that at this loading in the middle of the percolation curve there were a larger number of conductive networks that were either newly formed or beneficially aligned due to the microwave irradiation curing procedure. It is important to note that the trend of the relative rate of temperature increase for each loading during microwave curing matched the trend of the percentage increase of conductivity from their oven-cured counterparts. These findings and observations proved useful in future studies in this work to optimize the microwave settings to produce the lowest loading samples at the highest conductivity.

**Figure 6:**
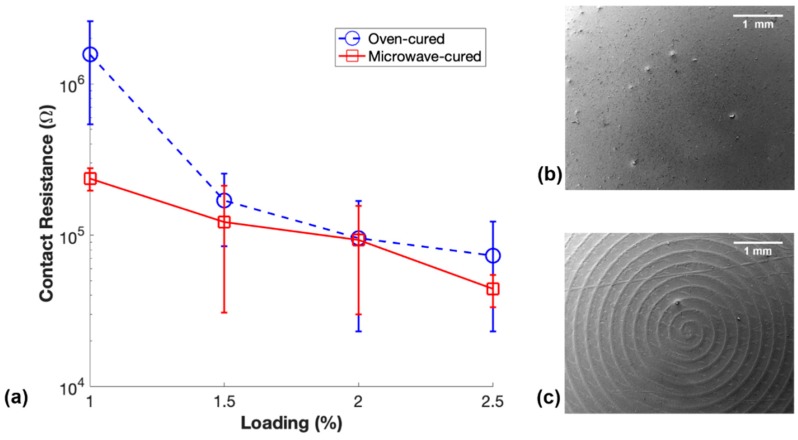
(**a**) Contact resistance of m-PDMS/CNT and t-PDMS/CNT for each conductive loading, (**b**) SEM image of m-PDMS/CNT surface cured in contact with glass mold, and (**c**) SEM image of t-PDMS/CNT cured in contact with aluminum mold.

Another notable finding in this study was the difference in the contact resistance of these conductive elastomeric nanocomposites. Contact resistance is a commonly unreported issue with these materials that is usually overcome in the field with the use of silver paste, or in our case, silver epoxy and copper tape. Results of the measured contact resistance of the m-PDMS/CNT and t-PDMS/CNT samples is shown in Figure 6, along with SEM image of the surfaces of the samples that were in contact with their respective molds during fabrication.

The general trend in Figure 6a shows that m-PDMS/CNT samples had a lower average contact resistance than t-PDMS/CNT, likely due to surface effects of the molds and potentially CNT settlement. The error bars are large due to a relatively inconsistent method used to cut the top expanded surface of the samples by hand with a razor blade. However, the average contact resistances for 1 wt.%, 1.5 wt.%, 2 wt.%, and 2.5 wt.% m-PDMS/CNT were 84.9%, 28.4%, 2.8%, and 39.8% less, respectively, than the average contact resistance of t-PDMS/CNT. This can most likely be explained by the surface effects on the samples introduced by the glass and aluminum molds. The SEM image displayed in Figure 6b shows the smooth surface of 1 wt.% m-PDMS/CNT advantageous for smooth consistent contact with the copper plate electrodes. In contrast, Figure 6c shows an SEM image of the spiral-shaped uneven surface of 1 wt.% t-PDMS/CNT as a result of the toolpath of the CNC milling machine used to create the aluminum mold. These differences in surface topography of m-PDMS/CNT and t-PDMS/CNT may have led to the differences in average contact resistance when loaded between the two electrodes.

### 3.4. Impacts of Microwave Curing Parameters

**Figure 7:**
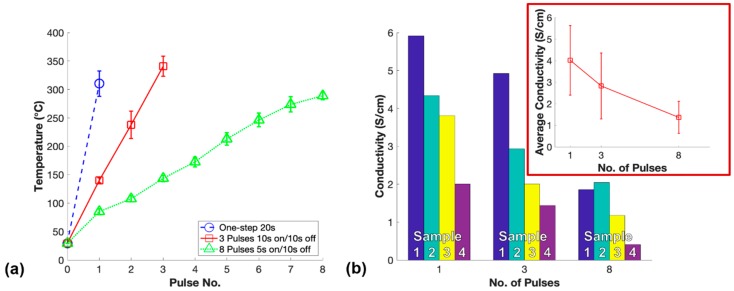
Pulse study—(**a**) Average temperature measurements between pulses of 50% microwave power used to cure 1 wt.% PDMS/CNT and (**b**) conductivity of each set of samples for the varying number of pulses used to fabricate 1 wt.% m-PDMS/CNT.

In order to isolate the microwave curing parameters and explore their impact on the conductivity of m-PDMS/CNT, the lowest conductive loading of material (1 wt.%) was chosen for further microwave curing investigation. The first investigation performed was the pulse study to examine whether pulsing the microwave on and off had an impact on the conductivity of the fabricated nanocomposites. Each sample was cured in 2 min or less to effectively negate settlement effects and isolate the microwave-induced CNT alignment effects to observe their impact on the conductivity of the samples. The temperature and conductivity results for the pulse study varying the number of 50% power microwave pulses to cure 1 wt.% PDMS/CNT are shown in Figure 7.

The temperatures of the samples during microwave curing rose quicker for the samples cured with fewer pulses as longer periods of continuous microwave irradiation exposure were applied to fully cure the nanocomposites. In this study, one-step microwave curing of PDMS/CNT nanocomposites produced the highest average conductivity followed by three pulses and lastly eight pulses as shown in Figure 7b. The faster increase in temperature during the microwave curing process corresponded with the higher average conductivity of the samples. The increased overall time of the microwave pulsed off during the curing process allowed polymerization to continue during this time due to substantial heat remaining within the resin. The partially cured resin while not being exposed to microwave irradiation likely increased the viscosity of the resin which in turn resisted the alignment mechanism advantageous for enhanced conductivity when exposed to microwaves later. Therefore, the CNT alignment effects induced by microwave exposure were highest for one-step microwave curing resulting in the highest average conductivity. Notably, the conductivity of each fabricated sample (Figure 7b) exhibited the influence of 15 min of settlement effect within the PDMS/CNT prepolymer that occurred between the fabrication of each set of samples. This settlement effect between various sets of samples explained the large error bars seen for the average conductivities; however, the trend for each set of samples was fairly consistent indicating the significance of one-step microwave curing to fabricate the highest conductivity nanocomposite.

**Figure 8:**
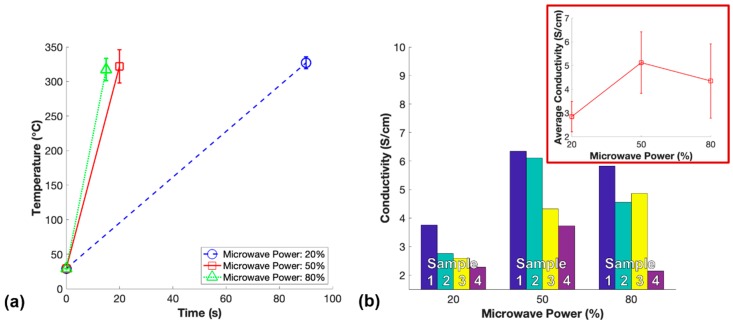
Power study: (**a**) Average temperature measurements after microwave curing nanocomposites with 1 wt.% CNT for one-step at variable microwave powers, and (**b**) conductivity of each set of samples for varying microwave powers used to fabricate 1 wt.% m-PDMS/CNT.

The power study was performed using one-step microwave irradiation to isolate the microwave power setting of the unmodified commercial microwave as a parameter that could impact the conductivity of m-PDMS/CNT. While the CNT alignment effects generated by microwave irradiation have not been well understood, it is important to investigate all possibilities that may impact this alignment. In this work the potential CNT alignment was measured as bulk conductivity of the fabricated nanocomposites. Therefore, the impact of 20%, 50%, and 80% microwave power during one-step microwave curing of 1 wt.% PDMS/CNT prepolymer was investigated and the results are shown in Figure 8.

Notably, the power of the unmodified commercial microwave when ran at any power less than 100% was controlled by pulsing on and off the magnetron. At 20% microwave power, the temperature increase of 1 wt.% PDMS/CNT during curing was significantly slower than 50% and 80% power. The progressive increase of the viscosity of the resin possibly prevented CNT alignment over this longer curing time, as well as the potential magnetron pulsing off allowing the resin to cure while not experiencing alignment effects endowed by microwave irradiation could explain this finding. Surprisingly, m-PDMS/CNT cured at 80% power had a slightly lower average conductivity than the samples cured at 50% power. This could be due to an enhanced rate of polymerization slowing the CNT alignment process through thixotropic effects or rapid curing locking the orientation of the CNTs in place before fully aligning. On the contrary, it is possible the polymerization had not fully propagated through the sample by the time the temperature had built up to above 300 °C, where the microwave exposure was stopped to prevent the samples from ripping apart due to intense thermal shock. Thus, the sample may have finished curing after microwave exposure ceased and the 5 s shorter time exposed to microwave irradiation as a resin would result in less CNT alignment and a slightly lower average conductivity. However, the average conductivity of m-PDMS/CNT cured at 50% power and 80% were reasonably close, so they both may have reached near maximum conductivity enhancement for microwave curing 1 wt.% PDMS/CNT. Regardless, one-step microwave irradiation at 50% power was found to be the optimal parameters to fabricate 1 wt.% m-PDMS/CNT with the highest conductivity.

### 3.5. Effects of Curing Methods on Electrical Properties

**Figure 9:**
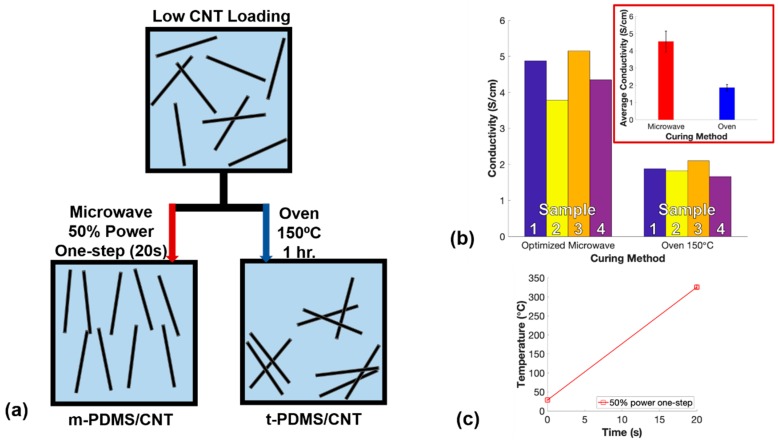
Optimized comparison—(**a**) Schematic showing improved CNT dispersion and alignment for nanocomposites cured with optimal microwave parameters, (**b**) conductivity of each set of samples for optimal m-PDMS/CNT and t-PDMS/CNT, and (**c**) average temperature measurements after microwave curing 1 wt.% PDMS/CNT for one-step at 50% microwave power.

After optimizing the microwave curing parameters to obtain the highest conductivity of 1 wt.% m-PDMS/CNT, it was imperative to compare the optimal microwave-cured samples with oven-cured samples to quantify the improvements made through the optimization process. For the final comparison, one-step 50% power microwave irradiation was used to fabricate 1 wt.% m-PDMS/CNT while simultaneously fabricating 1 wt.% t-PDMS/CNT in an oven at 150 °C. These optimized microwave settings were believed to improve the alignment of CNTs within the nanocomposite further increasing the conductivity of the nanocomposite (Figure 9a). The conductivity of each sample produced from both curing methods and the results of the temperatures that the PDMS/CNT encountered from microwave curing are shown in Figure 9b,c, respectively.

The microwave temperature results in this study (Figure 9c) matched the temperature results shown in Figure 7a and Figure 8a for the same microwave parameters and the same 1 wt.% CNT loading in the cured nanocomposites. These results confirmed that relatively consistent microwave curing temperatures were produced between batches of 1 wt.% m-PDMS/CNT. The average conductivities of the optimal m-PDMS/CNT and t-PDMS/CNT were 4.54 S/cm and 1.87 S/cm, respectively, indicating a 142.8% improvement of conductivity for the microwave-cured nanocomposites using the optimized microwave parameters. This was a huge improvement from the initial 44.8% conductivity enhancement demonstrated in the first comparison using 20% microwave power and 3 pulses of 30 s. The findings in this study demonstrated the importance of optimizing commercial microwave settings to achieve the largest conductivity enhancements for CNT-based nanocomposites fabricated via microwave curing.

## 4. Conclusions

In this paper, microwave irradiation was demonstrated to be an extremely time-efficient method to cure cylindrical conductive samples of PDMS/CNT in as little as 15 s. The microwave curing parameters, including the number of pulses and microwave power, were optimized to produce the highest conductivity samples of the lowest conductive loading of PDMS/CNT synthesized in this study. The optimal microwave settings were found to be one-step exposure at 50% microwave power. Experimental results demonstrated that nanocomposites with 1 wt.% CNT loading was able to increase the overall electrical conductivity of the nanocomposite by 142.8% over thermally cured samples due to reducing the settlement effect of the nanoparticles and likely improving CNT alignment. This impressive conductivity enhancement and time-efficient fabrication method are highly desirable in many fields using electrically conductive CNT-based nanocomposites. Microwave curing has a promising future as a viable curing method for carbon-based elastic nanocomposites.
